# Targeting JAK/STAT Signaling Antagonizes Resistance to Oncolytic Reovirus Therapy Driven by Prior Infection with HTLV-1 in Models of T-Cell Lymphoma

**DOI:** 10.3390/v13071406

**Published:** 2021-07-20

**Authors:** Shariful Islam, Claudia M. Espitia, Daniel O. Persky, Jennifer S. Carew, Steffan T. Nawrocki

**Affiliations:** 1 Division of Translational and Regenerative Medicine, Department of Medicine, The University of Arizona Cancer Center, Tucson, AZ 85724, USA; shariful@arizona.edu (S.I.); espitiac@arizona.edu (C.M.E.); jcarew@arizona.edu (J.S.C.); 2 Division of Hematology and Oncology, Department of Medicine, The University of Arizona Cancer Center, Tucson, AZ 85724, USA; dpersky@uacc.arizona.edu

**Keywords:** reovirus, oncolytic virus, T-cell lymphoma, HTLV-1, Reolysin, Pelareorep

## Abstract

Human T-cell leukemia virus type 1 (HTLV-1) is a retrovirus that infects at least 10 million people worldwide and is associated with the development of T-cell lymphoma (TCL). The treatment of TCL remains challenging and new treatment options are urgently needed. With the goal of developing a novel therapeutic approach for TCL, we investigated the activity of the clinical formulation of oncolytic reovirus (Reolysin, Pelareorep) in TCL models. Our studies revealed that HTLV-1-negative TCL cells were highly sensitive to Reolysin-induced cell death, but HTLV-1-positive TCL cells were resistant. Consistent with these data, reovirus displayed significant viral accumulation in HTLV-1-negative cells, but failed to efficiently replicate in HTLV-1-positive cells. Transcriptome analyses of HTLV-1-positive vs. negative cells revealed a significant increase in genes associated with retroviral infection including interleukin-13 and signal transducer and activator of transcription 5 (STAT5). To investigate the relationship between HTLV-1 status and sensitivity to Reolysin, we infected HTLV-1-negative cells with HTLV-1. The presence of HTLV-1 resulted in significantly decreased sensitivity to Reolysin. Treatment with the JAK inhibitor ruxolitinib suppressed STAT5 phosphorylation and expression of the key anti-viral response protein MX1 and enhanced the anti-TCL activity of Reolysin in both HTLV-1-positive and negative cells. Our data demonstrate that the inhibition of the JAK/STAT pathway can be used as a novel approach to antagonize the resistance of HTLV-1-positive cells to oncolytic virus therapy.

## 1. Introduction

T-cell lymphomas (TCLs) are a heterogeneous group of lymphoid malignancies [1,2]. Adult T-cell lymphoma/leukemia (ATL) is associated with human T-cell leukemia virus type 1 (HTLV-1) infection and constitutes 1–2% of cases of TCL in North America. However, HTLV-1 infects approximately 10–20 million people worldwide and is endemic in several countries, including southern Japan, the Caribbean islands, and Central and Latin America [3,4]. Patients with ATL exhibit a poor prognosis and are frequently resistant to conventional chemotherapy. As there are limited effective treatment options for patients with ATL, new therapeutic strategies are desperately needed. HTLV-1 infection triggers oncogenic signaling in transfected TCL cells [5,6]. The HTLV-1 protein Tax plays a key role in the activation, proliferation, and transformation of T cells through the activation of various genes including interleukin-2 (IL-2) and interleukin-13 (IL-13) [7,8]. IL-2 induces T-lymphocyte proliferation via the JAK/STAT signaling pathway, which is constitutively active in most cases [9]. IL-13 also exerts proliferative and anti-apoptotic functions and is associated with leukemogenesis [8]. Collectively, these studies demonstrate that the HTLV-1 Tax protein induces proliferation and transformation and is essential for the pathogenesis of HTLV-1-induced TCL.

Oncolytic virus therapy has demonstrated significant potential in a variety of tumor types, and this has resulted in several viruses progressing to clinical testing [10,11]. Reovirus is a non-enveloped, double-stranded RNA virus that is classified as an orphan virus due to the lack of association with any known human disease [12,13]. Previous studies have demonstrated that reovirus (type 3 Dearing strain) selectively infects and kills malignant cells without harming normal cells, which makes it appealing for therapeutic development [14,15,16]. Consistent with this idea, a proprietary formulation of oncolytic reovirus (Reolysin, Pelareorep) has been developed and tested in numerous clinical trials. Importantly, these studies established that Reolysin was very well tolerated and exhibited significant efficacy against a variety of cancer types, particularly when given in combination with standard of care regimens [17,18]. Here, we investigated the activity of Reolysin in models of TCL.

Given the prominent role of HTLV-1 in some TCLs, we hypothesized that oncolytic reovirus therapy may have differential sensitivity in HTLV-1-positive vs. negative cells. Our investigation revealed that HTLV-1-negative TCL cells were highly susceptible to reovirus infection and replication, which resulted in decreased cell viability. In contrast, HTLV-1-positive TCL cells were resistant to productive reovirus infection and did not undergo cell death following Reolysin treatment. We also determined that HTLV-1-positive TCL cells displayed significantly upregulated STAT5 phosphorylation compared to HTLV-1-negative cells, suggesting that they exhibit constitutive anti-viral defense mechanisms. Importantly, treatment with the JAK inhibitor ruxolitinib enhanced sensitivity to Reolysin in both HTLV-1-positive and negative TCL cells. Taken together, our findings provide the foundation for future clinical studies testing Reolysin in combination with JAK inhibitors for patients with TCL.

## 2. Materials and Methods

### 2.1. Cells and Cell Culture

KARPAS-299 cells were obtained from the European Collection of Authenticated Cell Cultures (ECACC, Salisbury, UK). HuT-78, HuT-102, and MJ cells were purchased from American Type Culture Collection (ATCC, Manassas, VA, USA). TCL cells were cultured with medium supplemented with 10% FBS at 37 °C with 5% CO_2_. HTLV-1-positive HuT-78 and KARPAS-299 cells were created by transfecting cells with HTLV-1 virus isolated from HuT-102 cells. Cell lines were authenticated by the source banks using short tandem repeat (STR) DNA profiling techniques. Cell numbers were counted before initiating each experiment via automated trypan blue exclusion with the assistance of a Vi-Cell XR system (Beckman-Coulter, Brea, CA, USA).

### 2.2. Chemicals and Reagents

Ruxolitinib was purchased from SelleckChem (Houston, TX, USA). Reolysin was kindly provided by Oncolytics Biotech, Inc. (Calgary, AB, Canada). Reovirus was initially quantified by Oncolytics Biotech, Inc. to meet Food and Drug Administration (FDA) standards for clinical administration to humans. Plaque assays confirmed viral titers. All experiments were performed with the clinical formulation of reovirus (Reolysin). Propidium iodide (PI) and 3-(4,5-dimethylthiazol-2-yl)-2,5-diphenyltetrazolium bromide (MTT) were purchased from Sigma (St. Louis, MO, USA).

### 2.3. Quantification of Drug-Induced Cytotoxicity

Cell viability was assessed by MTT assay. TCL cells were cultured in 96-well plates at a density of 0.1 × 10^6^ cells per ml with 200 µL of media per well and were treated with the indicated concentrations of drugs for 72 h. After drug treatment, MTT was added, and viability was quantified using a microplate reader. The pro-apoptotic effects of ruxolitinib and Reolysin were quantified by propidium iodide staining and fluorescence activated cell sorting (PI-FACS) analysis of sub-G0/G1 DNA and quantification of active caspase-3 positive cells by flow cytometry using a commercial kit (BD Biosciences, San Jose, CA, USA).

### 2.4. Transmission Electron Microscopy

HuT-78, KARPAS-299, HuT-102 and MJ cells were treated with 90 plaque forming units (PFU)/cell Reolysin for 48 h and processed for transmission electron microscopy as previously described [19]. The percentage of cells positive for reovirus infection was determined by manual counting of cells using transmission electron microscopy images. Approximately 200 cells were counted in independent groups of 60–70 cells each.

### 2.5. Immunoblotting and Antibodies

Protein identification by immunoblotting was performed following an optimized protocol as previously described [2]. Antibodies were obtained from the following sources: anti-p-STAT5 and JAM-A (Abcam, Cambridge, MA, USA) and anti-STAT5, MX1 and ß-actin from Cell Signaling (Danvers, MA, USA).

### 2.6. RNA Isolation and Expression Arrays

Total RNAs were isolated from HuT-78, KARPAS-299, HuT-102 and MJ cells using the RNeasy Plus Mini Kit (Qiagen, Germantown, MD, USA) and treated with the TURBO DNA-free Kit (Applied Biosystems, Foster City, CA, USA). A total of 300 ng of total RNA per sample was amplified and hybridized to GeneChip Human Gene 1.0 ST arrays (Affymetrix, Inc., Santa Clara, CA, USA) according to the manufacturer’s instructions. Affymetrix CEL files were imported into Partek Genomics Suite 6.4 (Partek Inc., St. Louis, MO, USA) using the default Partek normalization parameters and the robust multiarray average analysis adjusted for probe sequence and guanine and cytosine robust multiarray average content. Data normalization was performed across all arrays using quantile normalization. Finally, significantly expressed genes between HTLV-1-negative and HTLV-1-positive cells were searched in the DAVID database system to identify the most affected pathways as previously described [20]. Data from HTLV-1-positive vs. negative cells were averaged using Partek Genomics Suite 6.4 to determine significant differences in gene expression.

### 2.7. Quantitative Real-Time Polymerase Chain Reaction

Complementary DNA (cDNA) from parental KARPAS-299, HuT-78, MJ, HuT-102 and HTLV-1 transfected KARPAS-299 and HuT-78 cells were used for relative quantification of gene expression by real-time polymerase chain reaction (RT-PCR) analyses. First-strand cDNA synthesis was performed with 1 mg RNA in a 20 µL reaction mixture using the high-capacity cDNA Reverse Transcription Kit (Applied Biosystems, Foster City, CA, USA). *IL-13*, *STAT5*, *HTLV-1 (rex, tax)* transcripts were amplified using commercially available TaqMan^TM^ Gene expression assays (Applied Biosystems, Foster City, CA, USA). Relative gene expression was calculated with the 2^ΔΔ^^Ct^ method [21]. *ß-actin* was used as a housekeeping control gene. For quantification of reovirus by qRT-PCR, total RNA was isolated using RNeasy^®^ Plus Mini Kit (Qiagen, Germantown, MD, USA). First-strand cDNA synthesis was performed using the High-Capacity cDNA Reverse Transcription Kit (ThermoFisher, Waltham, MA, USA). Reovirus transcripts were amplified by qRT-PCR. Primers were designed to span the Reovirus type 3 (Dearing strain) S3 segment. Primers and TaqMan probe sequences are as follows: Forward primer: 5′-tgtatgacgctggctctacc-3′; Reverse primer: 5′-cgtccacctcacatccatag-3′; Probe: 5′Fam-cgctccaacactgtcagcgga-3′Tamra. Fold change was calculated using the comparative Ct (2^−ΔΔCt^) method. *ß-actin* was used as housekeeping gene.

### 2.8. Statistical Analyses

Statistical significance between samples was determined by using the Student’s *t*-test or the 2-way ANOVA multiple comparison test as appropriate. Differences were considered significant in all experiments at *p* < 0.05 with two-sided comparisons. All experiments were performed with a minimum of *n* = 3 from independent experiments.

## 3. Results

### 3.1. Reolysin Decreases TCL Cell Viability and Induces Apoptosis in HuT-78 and KARPAS-299 Cells

**Figure 1:**
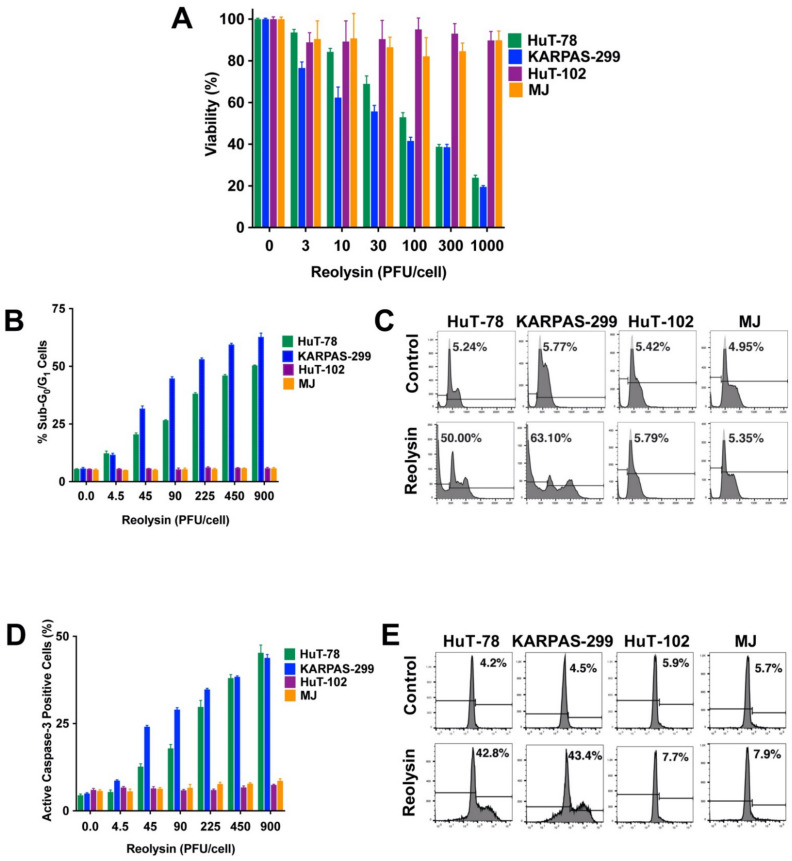
Reolysin exhibits anticancer efficacy against HuT-78 and KARPAS-299 TCL cells. (**A**) Effects of Reolysin on TCL cell viability. TCL cells were treated with the indicated concentrations of Reolysin for 72 h. Cell viability was measured by MTT assay. Mean ± SD, *n* = 3; (**B**,**C**) Reolysin induces DNA fragmentation in HuT-78 and KARPAS-299 TCL cells. TCL cells were treated with the indicated concentrations of Reolysin for 48 h. Induction of apoptosis was measured by PI-FACS analysis. Representative histograms are presented in untreated (Control) cells and cells treated with 900 PFU/cell Reolysin for 48 h. (**D**,**E**) Reolysin induces active caspase-3 in HuT-78 and KARPAS-299 cells. Cells were treated with the indicted concentrations of Reolysin for 48 h, and active caspase-3 levels were determined by active caspase-3 fluorescent staining followed by flow cytometry. Representative histograms are presented in untreated (Control) cells and cells treated with 900 PFU/cell Reolysin for 48 h. Mean ± SD, *n* = 3.

TCLs are a heterogenous group of lymphoid malignancies that are very aggressive and exhibit a poor prognosis. With the goal of developing a new therapeutic approach, we investigated the replication ability and efficacy of oncolytic reovirus in models of TCL. We initially tested the anti-TCL activity of Reolysin in a panel of TCL cell lines (KARPAS-299, HuT-78, HuT-102, and MJ). Reolysin displayed significant anticancer activity against two TCL cell lines (KARPAS-299 and HuT-78), while the other two models (HuT-102 and MJ) demonstrated significant resistance to Reolysin (Figure 1A). We next evaluated whether the induction of apoptosis stimulated by Reolysin treatment was also altered in the TCL cell lines. Consistent with the cell viability data, Reolysin induced apoptosis as measured by PI-FACS (Figure 1B,C) and active caspase-3 (Figure 1D,E) analysis selectively in the KARPAS-299 and HuT-78 TCL cell lines.

### 3.2. Oncolytic Reovirus Displays Productive Infection in HuT-78 and KARPAS-299 TCL Cells

**Figure 2:**
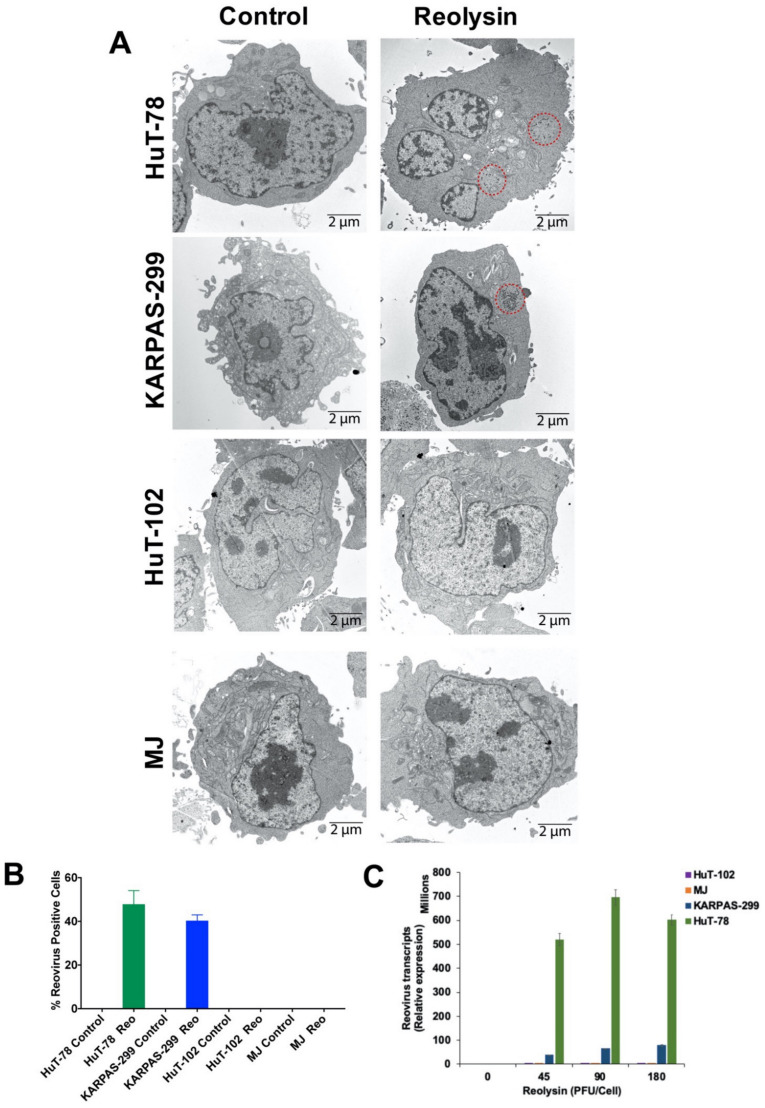
HuT-78 and KARPAS-299 TCL cells are sensitive to oncolytic reovirus replication. (**A**) Oncolytic reovirus replicates in HuT-78 and KARPAS-299 cells. HuT-78, KARPAS-299, HuT-102 and MJ cells were treated with 90 PFU/cell Reolysin for 48 h. Reovirus accumulation was visualized by transmission electron microscopy. Red circles indicate reovirus accumulation. Representative images are shown. (**B**) Quantification of reovirus in TCL cells. The percentage of reovirus-positive cells was counted in approximately 200 cells per cell line using transmission electron microscopy. No reovirus-positive cells were observed in the HTLV-1-positive HuT-102 and MJ cell lines. Mean ± SD. (**C**) Quantification of reovirus transcripts by qRT-PCR. TCL cell lines were treated with the indicated concentrations of Reolysin for 48 h. Relative levels of reovirus were determined by comparing 48 h Reolysin-treated samples with Controls for each cell line by qRT-PCR. Mean ± SD, *n* = 3.

To further investigate the differences in the sensitivity of our TCL cell lines to oncolytic reovirus therapy, we measured reovirus replication by transmission electron microscopy. In agreement with our cell viability and apoptosis data, reovirus replication was only observed in the HuT-78 and KARPAS-299 TCL cell lines (Figure 2A). The percentage of cells that displayed reovirus infection was determined by manual counting of reovirus-positive cells from transmission electron microscopy images. Significant reovirus accumulation was observed in the HuT-78 and KARPAS-299 cell lines, but no detectable viral particles were found in the HuT-102 or MJ TCL cell lines (Figure 2B). We further confirmed these results by evaluating reovirus transcripts by qRT-PCR in the four TCL cell lines. Consistent with our imaging data, only the HuT-78 and KARPAS-299 cells exhibited significant levels of reovirus transcripts (Figure 2C). The reovirus-susceptible HuT-78 and KARPAS-299 cell lines are HTLV-1-negative, while the HuT-102 and MJ cell lines are positive for HTLV-1 infection. It is possible that pre-infection with HTLV-1 may promote resistance to additional infection via oncolytic reovirus.

### 3.3. HTLV-1-Positive TCL Cells Display Significantly Upregulated IL-13 and STAT5 Expression

**Figure 3:**
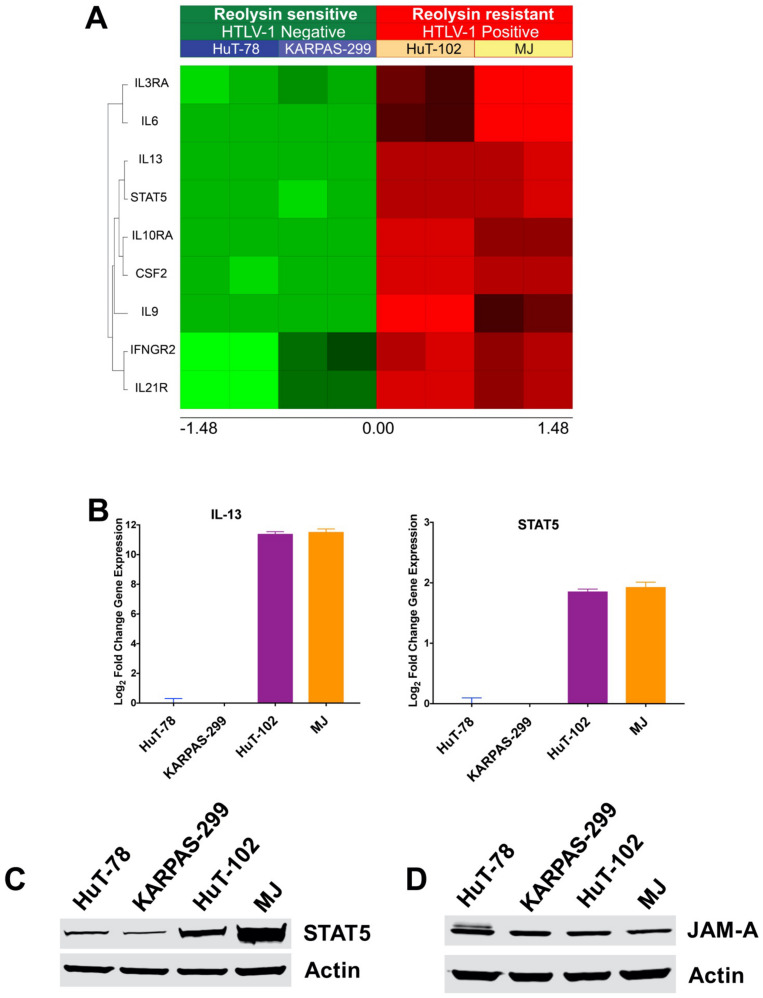
HTLV-1-positive TCL cells exhibit upregulated IL-13 and STAT5 expression. (**A**) Transcriptome analysis of HTLV-1-negative (HuT-78 and KARPAS-299) and HTLV-1-positive (HuT-102 and MJ) cell lines reveals significant alterations in JAK/STAT pathway-related genes including upregulation of IL-13 and STAT5. Gene expression changes were determined using Affymetrix expression arrays. Significantly upregulated genes are illustrated in the heatmap. (**B**) IL-13 and STAT5 levels are increased in HTLV-1-positive TCL cells. IL-13 and STAT5 expression was measured by quantitative real-time PCR in TCL cells. Mean ± SD, *n* = 3. (**C**) STAT5 expression is upregulated in HTLV-1-positive TCL cells. Immunoblotting determined that HuT-102 and MJ cells display increased expression of STAT5 compared to HTLV-1-negative TCL cells. (**D**) JAM-A expression is similar in 4 TCL cell lines. JAM-A levels were measured by immunoblotting.

To better understand the differences between HTLV-1-positive and negative TCL cell lines, we conducted transcriptome analysis on the models. Notably, genes involved in the Janus kinases (JAK) and Signal Transducers and Activators of Transcription (STAT) signaling pathway were significantly altered in both the HuT-102 and MJ cell lines. This may contribute to resistance to oncolytic reovirus therapy by promoting conditions that hinder viral replication. These genes have also been associated with the HTLV-1 Tax protein in that the presence of Tax drives their expression [7,22]. Consistent with HTLV-1 infection, *IL-13* and *STAT5* were significantly upregulated selectively in the HuT-102 and MJ cell lines (Figure 3A). This result was confirmed by qRT-PCR analysis (Figure 3B). A complete list of genes that were significantly altered in HTLV-1-positive (HuT-102, MJ) vs. HTLV-1-negative (HuT-78, KARPAS-299) cells is provided in Supplementary Table S1. Further analysis demonstrated that STAT5 expression was also increased at the protein level in HTLV-1-positive cell lines (Figure 3C). The expression of the reovirus receptor junctional adhesion molecule-A (JAM-A) has previously been reported to be a key factor that regulates reovirus infection [23,24]. To determine if altered JAM-A expression was causing differential reovirus sensitivity, we measured its expression by immunoblotting. Significant JAM-A expression was detected in all of the TCL cell lines, indicating that low JAM-A expression was likely not a key factor driving resistance to reovirus in the HuT-102 and MJ cell lines (Figure 3D). Collectively, these results suggest a link between HTLV-1 infection, JAK/STAT pathway activation, and resistance to oncolytic reovirus replication.

### 3.4. HuT-78 and KARPAS-299 TCL Cells Are Susceptbile to HTLV-1 Infection

**Figure 4:**
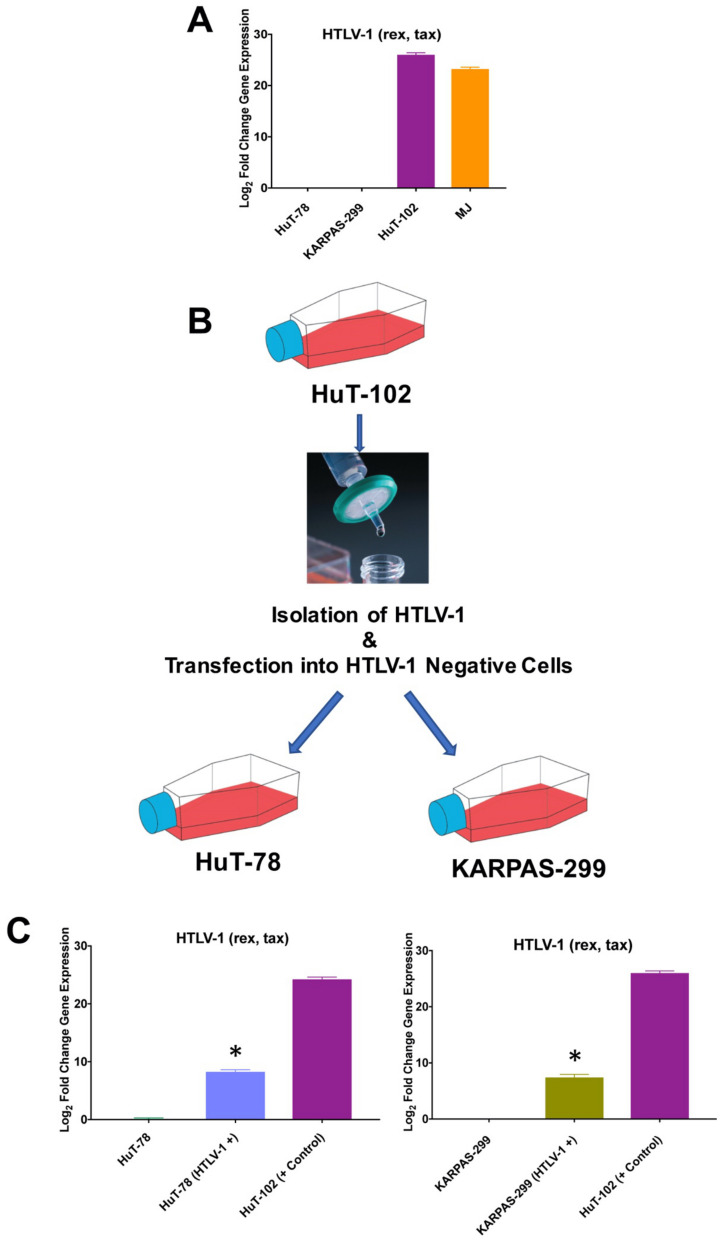
HTLV-1 isolated from HTLV-1-positive HuT-102 cells can infect other TCL cells. (**A**) Quantitative detection of HTLV-1 (rex, tax) by qRT-PCR in TCL cells. Mean ± SD, *n* = 3. (**B**) Transfection of HTLV-1-negative HuT-78 and KARPAS-299 cells by culture filtrate from HuT-102 cells. (**C**) Quantitative detection of HTLV-1 (rex, tax) by qRT-PCR in HTLV-1-negative and HTLV-1-infected models. HTLV-1-positive HuT-102 cells served as a positive control. Mean ± SD, *n* = 3. * Indicates a significant difference compared to non-infected cells, *p* < 0.05.

HuT-102 and MJ TCL cells have been previously characterized as exhibiting constitutive infection with HTLV-1 [25,26]. To confirm these findings, we evaluated HTLV-1 (rex, tax) by qRT-PCR in our TCL cell lines. Consistent with prior studies, HuT-102 and MJ demonstrated positive expression for HTLV-1, while HuT-78 and KARPAS-299 were determined to be negative for HTLV-1 infection (Figure 4A). Previous work has shown that HTLV-1 can be isolated from positive cells and then used to infect other cell lines to generate HTLV-1-positive models [27]. To produce HTLV-1-positive TCL models, we collected the filtrate from HTLV-1-positive HuT-102 cells and exposed the HTLV-1-negative HuT-78 and KARPAS-299 cells to the viral particles (Figure 4B). Two days after infection, qRT-PCR analyses were performed to detect the presence of HTLV-1 in the HuT-78 and KARPAS-299 cells. HuT-102 cells were used as a positive control. Significant levels of HTLV-1 (rex, tax) transcript were detected in the infected TCL models (Figure 4C).

### 3.5. Infection with HTLV-1 Promotes Resistance to Oncolytic Reovirus Therapy

**Figure 5:**
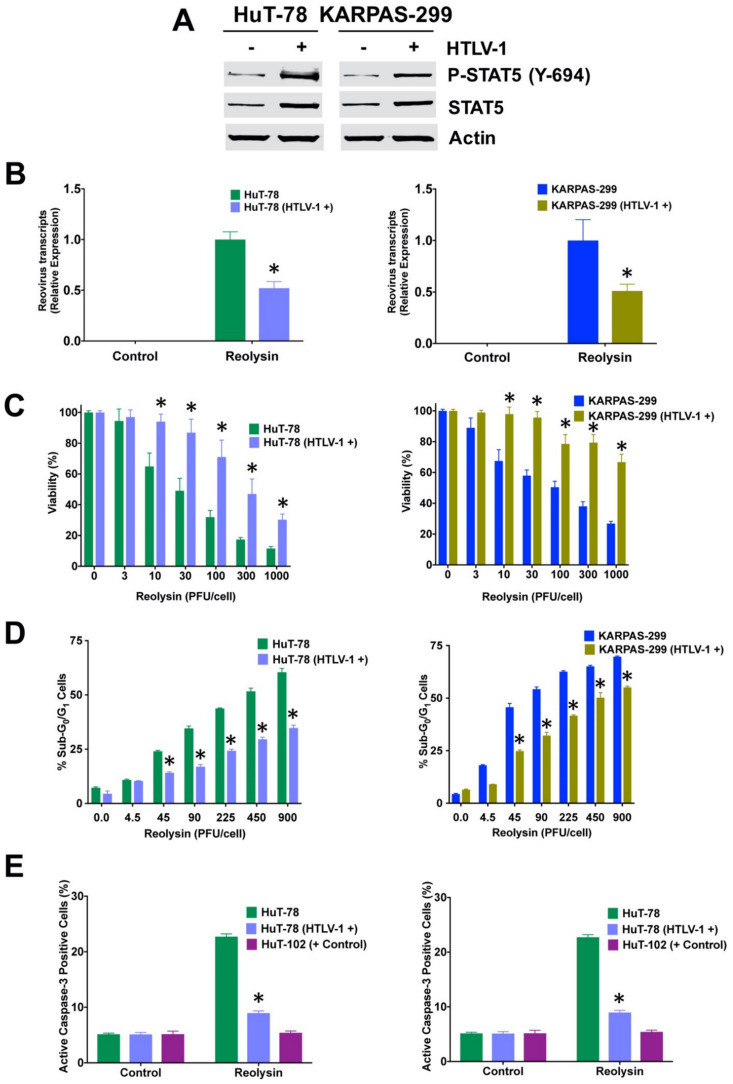
HTLV-1-infected TCL cells are less susceptible to Reolysin-induced cell death. (**A**) HTLV-1-infected TCL cells display upregulated expression of STAT5. Total and phospho-STAT5 levels were determined by immunoblotting in parental and HTLV-1-infected cells. (**B**) Quantification of reovirus levels in HTLV-1-negative and positive cells. Cells were treated with 180 PFU/cell Reolysin for 48 h. Levels of reovirus were quantified by qRT-PCR analysis. Transcript levels were quantified relative to Reolysin-treated HTLV-1-negative parental versions for each cell line. Mean ± SD, *n* = 3. (**C**) Reolysin is less effective at reducing TCL cell viability in the presence of HTLV-1. Parental and HTLV-1-positive cells were treated with the indicated concentrations of Reolysin for 72 h. Cell viability was measured by MTT assay. Mean ± SD, *n* = 3. (**D**) HTLV-1-transfected cells are significantly less sensitive to Reolysin-mediated apoptosis. Cells were treated with the indicated concentrations of Reolysin for 48 h and apoptosis was quantified by PI-FACS analysis. Mean ± SD, *n* = 3. (**E**) Reolysin-induced caspase-3 activation is reduced in the presence of HTLV-1. Cells were treated with 50 PFU/cell Reolysin for 48 h and active caspase-3 levels were quantified by flow cytometry. Mean ± SD, *n* = 3. * Indicates a significant difference between parental and HTLV-1-positive cells, *p* < 0.05.

To investigate the mechanistic relationship between HTLV-1 infection and sensitivity to oncolytic reovirus, we evaluated the anticancer activity of Reolysin in parental and HTLV-1-positive HuT-78 and KARPAS-299 cells. Consistent with our prior transcriptome analyses, exposure to HTLV-1 resulted in increased expression of STAT5 as well as STAT5 phosphorylation (Figure 5A). In agreement with the anti-viral effects of increased STAT5 activity, we also observed a significant decrease in reovirus transcripts in the HTLV-1-positive cells (Figure 5B). We next evaluated whether HTLV-1 infection would promote resistance to oncolytic reovirus therapy in TCL cell lines. Reolysin treatment was significantly more effective at reducing cell viability in the HTLV-1-negative cells (Figure 5C). In addition, the presence of HTLV-1 also blunted Reolysin-induced apoptosis as measured by PI-FACS analysis (Figure 5D) and the determination of active caspase-3 (Figure 5E).

### 3.6. Ruxolitinib Decreases STAT5 and MX1 Expression and Significantly Enhances the Anti-TCL Activity of Reolysin

**Figure 6:**
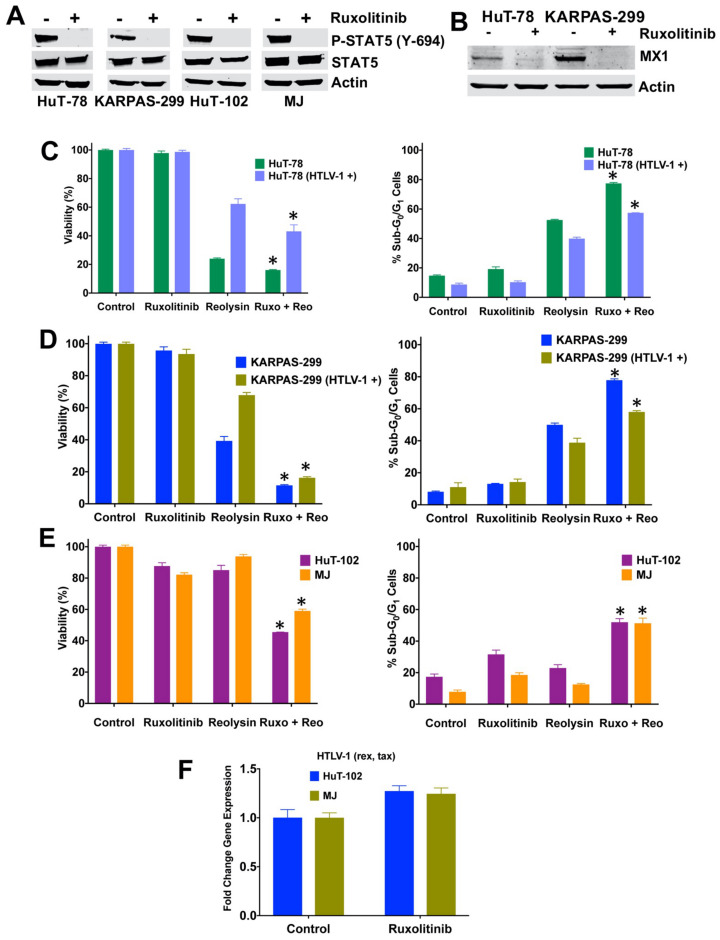
Ruxolitinib enhances the anti-TCL activity of Reolysin in TCL models regardless of HTLV-1 infection status. (**A**,**B**) Ruxolitinib treatment downregulates the expression of STAT5 phosphorylation and MX1 in TCL cells. TCL cell lines were treated with 10 μM ruxolitinib for 24 h and protein expression was measured by immunoblotting. (**C**,**D**) Ruxolitinib augments the efficacy of Reolysin in both the presence and absence of HTLV-1. HuT-78 and KARPAS-299 cells were treated with 10 μM ruxolitinib, 90 PFU/cell Reolysin (HuT-78) or 45 PFU/cell (KARPAS-299) and the combination for 72 h. Cell viability and apoptosis were assessed by MTT and PI-FACS assay, respectively. (**E**) Ruxolitinib augments the anti-TCL activity of Reolysin in HuT-102 and MJ cells. Cells were treated with 20 μM ruxolitinib, 1350 PFU/cell Reolysin and the combination for 72 h. Cell viability and apoptosis were assessed by MTT assay. For PI-FACS analysis, cells were treated with 15 μM ruxolitinib, 1350 PFU/cell Reolysin and the combination for 72 h. Mean ± SD, *n* = 3. * Indicates a significant difference between the combination and either monotherapy. (**F**) Effects of ruxolitinib on HTLV-1 expression. HTLV-1-positive TCL cells were treated with 20 μM ruxolitinib for 48 h. HTLV-1 (rex, tax) levels were measured by qRT-PCR. Mean ± SD, *n* = 4.

We next evaluated whether the pharmacological inhibition of JAK/STAT signaling with the JAK inhibitor ruxolitinib could downregulate STAT5 expression and augment Reolysin-mediated cell death. Parental TCL cells were treated with ruxolitinib for 24 h and STAT5 phosphorylation was determined by immunoblotting. Consistent with the drug’s mechanism of action, ruxolitinib dramatically inhibited STAT5 phosphorylation (Figure 6A). Myxovirus Resistance 1 (MX1) is a virus restriction factor that is upregulated in response to interferons and was determined to be significantly expressed in the HuT-78 and KARPAS-299 TCL cell lines. Importantly, its expression was also decreased by ruxolitinib treatment in the HuT-78 and KARPAS-299 cells (Figure 6B). Interestingly, MX1 expression was undetectable by immunoblotting in the HTLV-1-positive cells (HuT-102 and MJ), suggesting that chronic HTLV-1 expression may promote the loss of MX1. We next investigated whether treatment with ruxolitinib could be used as a strategy to augment the anti-TCL activity of Reolysin in HTLV-1-positive cells. Our assays demonstrated that ruxolitinib treatment significantly enhanced the anticancer activity of Reolysin in both the HuT-78 and KARPAS-299 models regardless of prior HTLV-1 infection (Figure 6C,D). We also evaluated whether the ruxolitinib and Reolysin combination would be effective in the pre-existing HTLV-1-positive HuT-102 and MJ TCL cell lines. Consistent with our other models, this combination significantly decreased cell viability and induced apoptosis in both cell lines with native HTLV-1 infections (Figure 6E). Finally, we also determined whether ruxolitinib treatment may increase HTLV-1 levels. We observed only a minor increase in HTLV-1 (rex, tax) transcripts by qRT-PCR (Figure 6F). Collectively, our data demonstrate that Reolysin in combination with ruxolitinib exhibits significant anti-TCL activity regardless of HTLV-1 status.

## 4. Discussion

Selective activity against malignant cells is one of the leading criteria that is prioritized in the development of novel anticancer agents. Accordingly, the therapeutic use of oncolytic viruses represents a promising anticancer approach due to their ability to selectively replicate in tumor cells without harming normal tissue. This distinctive replication profile has resulted in the development of several oncolytic viruses that have been tested in clinical trials for the treatment of various cancer types [10,28,29,30]. Reolysin (Pelareorep) is a clinical formulation of oncolytic reovirus that has progressed into many clinical trials and demonstrated promising anticancer activity [10,15,28,29,31,32]. These collective studies have established that Reolysin has an excellent safety profile and selectively replicates within the tumor cells of patients that have been treated with it. These findings underscore the need to gain a better understanding of the mechanisms that regulate oncolytic reovirus infectivity to facilitate its optimal clinical applications.

We previously determined that Reolysin possesses significant activity in TCL cell lines and, in particular, cells that have developed acquired resistance to histone deacetylase (HDAC) inhibitors [33]. Interestingly, the heightened sensitivity in the HDAC inhibitor-resistant cells was mediated by decreased expression of the anti-viral regulators interferon regulatory factor 1 (IRF1) and signal transducer and activator of transcription 1 (STAT1). This study revealed that certain cancer cell types may be hypersensitive to oncolytic virus therapy due to defective anti-viral response mechanisms that are either constitutively present or adaptively developed over the course of chemotherapeutic treatment. To build upon these foundational observations, we investigated the anticancer activity of Reolysin in additional models of TCL. Our study identified that cells with prior HTLV-1 infection demonstrated reduced sensitivity to Reolysin therapy due to the likely upregulation of anti-viral genes associated with HTLV-1 exposure. Our findings are consistent with a prior investigation in head and neck squamous cell carcinoma (HNSCC) models where oncolytic reovirus displayed blunted activity in HNSCC cells infected with human papillomavirus (HPV) [34]. While the authors proposed that resistance to reovirus in HPV-positive HNSCC cells could be due to decreased epidermal growth factor receptor (EGFR) expression and subsequent RAS activation, it is also possible that the presence of HPV upregulated the anti-viral response, as we observed in our study.

HuT-102 and MJ express high levels of HTLV-1 expression that could not be achieved through the transfection of HTLV-1-negative cell lines HuT-78 and KARPAS-299 with HTLV-1. While the HTLV-1-transfected cells displayed significant resistance to Reolysin, they did not yield the same level of resistance observed in the chronically infected HuT-102 and MJ models. These data indicate that sensitivity to Reolysin may also depend on the degree of HTLV-1 infection.

JAK/STAT signaling pathways, particularly STAT5, are frequently dysregulated and play an important role in the pathogenesis and disease progression of TCL [35,36,37]. Specifically, we discovered significant upregulation of IL-13 and STAT5 in HTLV-1-positive TCL cells, and this is representative of HTLV-1 infection in other models [7,8,22]. IL-13 is a cytokine with proliferative and anti-apoptotic functions that has been linked to tumorigenesis [7,38]. Our study suggests that STAT5 is a key regulator of reovirus sensitivity in TCL models. However, it is possible that the upregulation of IL-13 in HTLV-1-positive cells may also contribute to their reovirus-resistant phenotype. Additional studies evaluating the possible role of IL-13 in controlling reovirus sensitivity are warranted. While Reolysin has demonstrated significant clinical activity against many tumor types, these studies have also determined that its maximal potential is best achieved in combination with standard chemotherapy. In agreement with this idea, Reolysin has been shown to enhance the efficacy of a broad range of anticancer agents [10,11,39]. Here, we demonstrate that Reolysin in combination with the JAK inhibitor ruxolitinib displays significantly improved anti-TCL activity. Importantly, this therapeutic approach is beneficial in both HTLV-1-negative and positive cells, further suggesting that increased JAK/STAT pathway activation contributes to Reolysin resistance. Another investigation also demonstrated that reovirus could activate JAK/STAT signaling, which contributes to reduced reovirus infection in the brain [40]. In addition, targeting the JAK/STAT pathway has been reported to augment the anticancer activity of other oncolytic viruses [41,42,43,44]. We observed a minor increase in HTLV-1 transcripts following treatment with ruxolitinib. These results suggest that some caution may be required when administering JAK inhibitors in the HTLV-1-positive patient population. We plan to further investigate the potential of the Reolysin and JAK inhibitor combination in primary TCL patient specimens as well as in patient-derived xenograft mouse models. Collectively, these studies will provide the rationale to test this therapeutic approach in the clinic. Taken together, our findings indicate that screening patients for infectious viruses prior to initiating therapy with oncolytic viruses may be of value and also support further investigation of the safety and efficacy of the combination of Reolysin with JAK inhibitors.

## 5. Conclusions

Here, we demonstrate that HTLV-1-infected TCL cells promote resistance to oncolytic reovirus therapy through the upregulation of JAK/STAT signaling. Treatment with the JAK inhibitor ruxolitinib reduces this mechanism of resistance and augments the activity of Reolysin. Our findings provide the rationale for further testing of the combination of Reolysin and JAK inhibitors as a new approach for treating TCL.

## Data Availability

Not applicable.

## Supplementary Materials

The following are available online at https://www.mdpi.com/article/10.3390/v13071406/s1, Table S1: List of genes that are significantly altered 2.0-fold or greater in both HTLV-1-positive (HuT-102, MJ) cells vs. HTLV-1-negative (HuT-78, KARPAS-299) cells.
